# High Vitamin D Levels May Downregulate Inflammation in Patients with Behçet's Disease

**DOI:** 10.1155/2017/8608716

**Published:** 2017-06-04

**Authors:** Fahd Adeeb, Maria Usman Khan, Xia Li, Austin G. Stack, Joseph Devlin, Alexander D. Fraser

**Affiliations:** ^1^Department of Rheumatology, University Hospital Limerick, Limerick, Ireland; ^2^Graduate Entry Medical School, University of Limerick, Limerick, Ireland; ^3^Health Research Institute, University of Limerick, Ireland; ^4^Department of Medicine, Universiti Teknologi MARA, Sungai Buloh, Selangor, Malaysia; ^5^Department of Mathematics and Statistics, La Trobe University, Melbourne, VIC, Australia; ^6^Department of Nephrology, University Hospital Limerick, Limerick, Ireland

## Abstract

Vitamin D plays a significant role in the immune system modulation and may confer a protective role in autoimmune diseases. We conducted a case-control study to compare 25(OH)D levels in patients with BD who were managed at a regional rheumatology programme in the midwest region of Ireland compared to matched controls. Healthy controls were selected from the Irish health system and matched in 1 : 5 ratio for age, sex, and the month of the year. 25(OH)D levels <20 nmol/L were classified as deficient while levels between 20 and 40 nmol/L were classified as insufficient. Differences between groups were assessed using Mann–Whitney test and associations between cases and controls were expressed as odds ratios and 95% confidence intervals. Nineteen patients with BD were compared with 95 controls matched by age, sex, and month of blood draw. 25(OH)D levels were significantly higher in patients in BD than in matched controls (median values: 45 nmol/L versus 22 nmol/L, *p* < 0.005) and tended to be lower in patients with active disease than in those without (median values: 35 nmol/L (IQR: 22.75–47.25 nm/L) versus 50 nmol/L (IQR: 35–67 nmol/L), *p* = 0.11). Compared to controls, patients with BD were significantly less likely to have 25(OH)D deficiency or insufficiency (OR: 0.09, 95% CI: 0.03–0.28, *p* < 0.001). Our findings suggest a possible role for 25(OH)D in modifying the inflammatory response in BD and uncover a potential opportunity to assess whether correction of Vit D deficiency confers protective benefits.

## 1. Introduction

Behçet's disease (BD) is classified among vasculitides with a unique characteristic of having a predilection for both arteries and veins and manifesting with distinct clinical characteristics. Despite having a considerable geographical variation with the highest prevalence seen along the Silk Road, BD is seen throughout the world including European countries with latitudes above 45° north. Ireland is a country with a temperate maritime climate strongly influenced by the Atlantic Ocean due to its proximity at the periphery of northwest Europe at a high latitude of 51–55° north, with a relatively low sunshine exposure throughout the year [1].

Vitamin D (Vit D) is a major fat-soluble vitamin that is essential for normal immune function and a large body of evidence now implicates Vit D in the pathogenesis of several inflammatory diseases including BD [2–16]. The main source of Vit D is from the ultraviolet B- (UVB-) induced synthesis in the skin, which is then activated via two hydroxylation steps in the liver and kidney. Studies have shown that exposing 5% of the body surface in fair-skinned persons 2 to 3 times a week for 5 minutes of noontime summer sun exposure is equivalent to intake of 430 IU/d of Vit D, which satisfies the recommended Vit D intake of 200 IU/d for young adults [17]. The recommended intake is 400 IU/d for persons aged 51 to 70 years and 600 IU/d for those above 70 years of age [18]. A serum Vit D level of ≥30 ng/mL (75 nmol/L) is considered adequate to limit the release of parathyroid hormone or promote the intestinal absorption of calcium [19, 20].

Recent studies from Mediterranean countries and the Far East have uncovered potentially important links between Vit D and BD [9–16, 21–23]. Interestingly, the findings indicate an inverse correlation between Vit D and BD [9–15] with higher levels of Vit D correlating with lower levels of disease activity [12, 14, 16]. It is hypothesized that low levels of Vit D suppress T-regulatory cells (T*regs*) activity, cause skewing of the Th1/Th2 balance towards Th1, and impair endothelial function [10, 12]. However, not all studies have confirmed this association. Observational studies from Turkey and Tunisia found no significant differences between Vit D levels in cases with BD and matched healthy controls [21, 22], while contrastingly a study from Iran found higher Vit D levels among patients with BD [23].

The aim of our study was to determine the relationship between Vit D status and BD in an Irish population and assess whether Vit D levels varied according to disease activity. This study is the first to explore the association between Vit D and BD in a Northern European population.

## 2. Methods

### 2.1. Study Design

We conducted a case-control study of patients with BD and compared them to healthy controls selected from the Irish health system. Cases included all patients with BD who were managed in a regional rheumatology programme at University Hospital Limerick, a tertiary centre located in the midwest region of Ireland. All cases satisfied the International Study Group Criteria for Behçet's Disease (ISGBD) or the International Criteria for Behçet's Disease (ICBD). Medical records and electronic data including clinical and laboratory parameters of all cases were reviewed. Exclusion criteria include other rheumatological or bone/skeletal diseases or other diseases with higher inflammatory status, history of chronic kidney disease or other chronic systemic diseases, malignancies, and limited physical activity. Patients were also excluded if they received Vit D supplementation or medications that interfered with Vit D metabolism including calcium supplements, cytotoxic drugs, anticonvulsants, bisphosphonates, or thyroxine, but not glucocorticoids and disease-modifying antirheumatic drugs (DMARDs). Active disease in BD was defined as the presence of any characteristic symptoms including mucocutaneous, visceral, ocular, or skin lesions necessitating initiation or dose increment of systemic steroids and/or immunosuppressive therapy. Healthy controls (*n* = 95) were selected from a register of patients in the health system from years 2013 and 2014. Blood samples from controls were matched with cases based on age, sex, and month of blood draw in a 1 : 5 ratio. Control subjects had normal concentrations of serum creatinine, haemoglobin, and serum albumin.

### 2.2. Vitamin D Assessment

Total serum 25-hydroxyvitamin D (25(OH)D) level was measured by using competitive chemiluminescence immunoassays (DiaSorin, Dietzenbach, Germany) with interassay coefficient of variation (CV) of 6.8% [24, 25]. Levels less than 20 nmol/L were defined as Vit D deficient and between 20 and 40 nmol/L as Vit D insufficient, with “normal” defined as levels more than 40 nmol/L. The study was approved by the local ethics committee and is in accordance with the Declaration of Helsinki.

### 2.3. Statistical Analysis

Vit D levels were not normally distributed; therefore, median and interquartile ranges were used to describe distributions between cases and controls. Nonparametric statistics (Mann–Whitney *U* test) were used to compare serum Vit D levels between patients and controls. Odds ratios (OR) and 95% confidence intervals (95% CI) were used to determine the likelihood of Vit D deficiency and Vit D insufficiency for cases versus controls using multivariate logistic regression. *p* values of <0.05 were considered statistically significant. Statistical analyses were performed using SPSS 22.0 software for Macintosh.

## 3. Results

A total of 19 Caucasian patients with BD were included in the study sample (5 males, 14 females, median age of 37.5 years, interquartile range (IQR): 24.3–51.2 years). 15 patients were in clinical remission while 4 patients had evidence of active disease. Baseline characteristics of the study population are reported in Tables 1 and 2. There were no statistically significant differences between cases and the control group with regard to age, sex, and time of blood draw. Disease duration varied between 0.25 and 30 years (mean: 8.07 years).

Median serum concentrations of 25(OH)D for patients with BD and controls were 45 nmol/L (IQR: 33–65 nmol/L) and 22 nmol/L (IQR: 15–31 nmol/L), respectively,* p* < 0.005. The median 25(OH)D levels also tended to be lower among patients with active BD as compared to those with inactive disease, 35 nmol/L (IQR: 22.75–47.25 nmol/L) and 50 nmol/L (IQR: 35–67 nmol/L), respectively, but this did not reach statistical significance (*p* value = 0.11) (Table 3). Similarly, the prevalence of 25(OH)D deficiency and insufficiency was significantly lower in BD compared to matched controls (0% versus 42% for 25(OH)D deficiency and 6% versus 36% for 25(OH)D insufficiency, *p* < 0.005).

In the unadjusted analysis, compared to controls, patients with BD were significantly less likely to have 25(OH)D deficiency or insufficiency with the unadjusted analysis odds ratio (OR) 0.10 (0.03, 0.30). With adjustment for age, sex, and month of blood draw, the relationship was even more robust (OR: 0.09 (0.03, 0.28), *p* < 0.001). Women were more likely than men to have Vit D deficiency (OR: 3.01, 95% CI: 1.10–8.26) (Table 4).

## 4. Discussion

In this Irish study, we report significant differences in the concentrations of Vit D levels among patients with BD compared to healthy controls from the health system. In keeping with the previous Iranian study [23], our study found significantly and substantially higher 25(OH)D levels in patients with BD than in those without. The magnitude of the difference was quite substantial and our analysis indicated that patients with BD were over 10-fold less likely to have Vit D deficiency compared to controls without BD. Moreover, our study also suggested that patients with active BD tended to have lower Vit D levels compared to those with inactive disease. Our findings suggest a possible role for 25(OH)D in modifying the inflammatory response in BD and uncover a potential opportunity to assess whether correction of Vit D deficiency confers protective benefits.

The finding of higher concentrations of Vit D in patients with BD compared to control patients was an unusual yet intriguing observation. The threshold value of Vit D considered to be adequate for human health is 50 nmol/L and therefore the majority of both BD patients and controls had levels that were below this threshold limit [26]. Notwithstanding this, cases with BD experienced significantly higher levels than controls without the disease. Even more intriguing is the observation that patients with active BD tended to have lower Vit D levels than those without. It is possible that higher levels of Vit D are immunomodulatory and downregulate inflammatory pathways commonly associated with BD and thereby are protective against disease flares. The small sample size clearly makes it difficult to demonstrate significance but our observations support the findings from other published studies [12, 14, 16] and suggest that serum concentrations of 25(OH)D in subjects with BD correlate inversely with disease activity.

Another interesting finding in our study is that the majority of subjects in the control group were either Vit D insufficient or deficient. Ireland is located at the high latitudes of 51–55° north and receives between 1100 and 1600 hours of sunshine each year [27]. Due to its geographical position in Northwest Europe and its proximity to the Atlantic Ocean resulting in a low-pressure system and moderation of the climate, Ireland is said to have a maritime climate where summers are not too hot and winters are not too cold. The average amount of rainfall in the western region of Ireland is up to 225 days a year while the cloud immersion almost completely covers the skies for more than 50 percent of the time [27]. These combined factors together with possible uses of sunscreens ultimately result in inadequate quantity and ineffective quality of UV irradiation that may explain why hypovitaminosis D abounds within the Irish population [28, 29].

Vit D contributes to the regulation of the proliferation, differentiation, and function of immune cells, both directly and indirectly [30–32]. The perspective role of Vit D as an immunoregulatory hormone stemmed from in vitro studies of its metabolism by immune cells in the early 1980s [33, 34] and was further supported by the works of Fritsche and Van Etten and their respective colleagues who identified the Vit D receptor (VDR) in the immune cells such as neutrophils, dendritic cells, and activated lymphocytes [35], proving that Vit D was potently able to modulate a range of immune cell functions [36], respectively.

Vit D has been shown to enhance the effect of dendritic cells, macrophages, and monocytes and has been demonstrated to downregulate cellular response to tumor necrosis factor-alpha (TNF-*α*), B-cell antibody production, and proinflammatory cytokines such as interleukin-1 (IL-1), IL-2, IL-6, IL-12, and interferon-gamma (IFN-*γ*) [37–42]. Its receptor, VDR, is a nuclear hormone receptor that regulates the p450 enzymes and interacts with various interconnected lipid metabolism and immune signaling pathways that have instrumental roles in immunity including peroxisome proliferator-activated receptor-alpha (PPAR-*α*), PPAR-*γ*, NF-*κ*B, p38 mitogen-activated protein kinase (MAPK), transforming growth factor-beta (TGF-*β*), and eicosanoid synthesis [43]. It is also a ligand-regulated transcription factor of many target genes, namely, the natural endogenous antimicrobial peptides (AMPs) cathelicidin and *β*-defensin: both crucial in shaping the gut microbiota [43], barrier defense, and maintaining the integrity of the gut mucosa [44, 45]. These overall immunoregulatory effects underline Vit D as a key mediator of innate and adaptive immunity.

In addition, there has also been a paradigm shift regarding the different functions of Vit D, including its essential role in the skin [46–51]. Skin cells such as keratinocytes and fibroblasts express VDR, an absolute prerequisite for regulation of genomic effects of Vit D and its other analogs [47]. A randomized, double-blind placebo-controlled study demonstrated that Vit D supplementation resulted in a significant improvement in parameters of diabetic foot ulcers at 12 weeks compared to placebo [48]. Other studies have demonstrated the positive effect of Vit D in tissue formation and remodeling [49–51]. The recurrent, relapsing, and remitting nature of Behçet's disease (BD), especially the orogenital ulcers and cutaneous manifestations, and the fact that wound healing is a dynamic process which involves phases of inflammation, tissue formation, and remodeling [52] implicate Vit D as possibly one of the key players in the pathogenesis of BD.

The Vit D pathway is now strongly implicated to be involved in the pathogenesis of inflammatory diseases such as inflammatory arthritis, lupus, and BD [2–16]. Furthermore, VDR gene polymorphisms have been observed to be significantly associated with an increased susceptibility risk in different BD populations [53–56]. Recently, Kahraman et al. demonstrated elevated numbers of circulating extracellular vesicles (EVs), novel mediators of distant cellular communication in BD patients, and observed significantly higher levels of cathelicidin in plasma of active BD patients, with two-thirds of them being associated with the EVs and cytokine production, correlating them with the disease activity [57].

Gut microbiota (GM) dysbiosis characterized by the reduction of the GM ecosystem diversity and composition has been implicated in the proinflammatory features of several autoimmune disorders including immunological diseases outside of the gut [58, 59], and this impaired microbiome-host interaction seems to play a role in the pathogenesis of BD [60–62]. Recently, Wang et al. in their comprehensive analysis of genome-wide host-microbiota associations discovered significant associations between gut microbial characteristics and the VDR gene [63]. In another study, Yuk et al. demonstrated cathelicidin as the link between autophagy and Vit D mediated innate immunity [64]. The relationship between autophagy, which not just maintains the gut intracellular homeostasis but also promotes immunity in symbiosis with the VDR, and the gut microbiota shapes the intestinal immune response and the disarray of this interaction may play a crucial part in the pathogenesis of BD.

We acknowledge some inherent limitations in the present study and consequently definitive conclusions are limited.* First*, the relatively small sample size of our cohort with BD and the variability in the duration of disease posed a limitation in our study. However, the number of patients in this study is an accurate reflection of the low prevalence of BD in this Northern European geographical region. Further studies including collaborative efforts involving other BD cohorts may provide a greater insight into this specific area.* Second*, our analysis may be confounded by other factors such as body mass index and the differences in the sun exposure of cases (and controls) which may vary according to lifestyle, the use of sunscreen, and overall health.* Third*, we were also unable to adjust for season variation in Vit D as the study was conducted over 10 months to coincide with outpatient visits. However, we attempted to account for this confounder in our analysis by limiting the selection of controls only to those who had Vit D levels assessed during the same time period.

The strength of our study is the comparison with a control population who were selected from patients within the health system based on age, gender, and timing of taking the sample. Despite insufficient and inconclusive evidence in the literature, there is a growing appreciation and basis to suggest that Vit D supplementation could represent an essential adjuvant treatment in active inflammatory patients who are also found to be deficient or insufficient of Vit D. The results of our study, despite its limitations, may provide some basis to support this hypothesis. Further intervention studies however are needed to prove a beneficial effect of Vit D supplementation among BD patients.

In summary, our study found evidence of elevated 25(OH)D levels in patients with BD compared to controls in the health system. Patients with BD were over 10-fold less likely to have Vit D deficiency adjusting for age, sex, and timing of blood samples. Vit D levels tended to be also lower among those with active disease than among those without. Our findings suggest that Vit D may be a potential suppressor of the inflammatory response in BD. The contribution of Vit D to the pathogenesis of BD and as a therapeutic intervention deserves further investigation. Studies of larger size are required to explore the hypothesis suggested here.

## Figures and Tables

**Table 1:** Demographics and clinical characteristics of BD patients and healthy controls.

Variables	BD patients (*n* = 19)	Controls (*n* = 95)	*p* value
Age (years)	37.5	37.4	0.924
Median (Q1–Q3)	(24.3–51.2)	(24.1–51.4)	

Female, *n* (%)	14 (73.68)	70 (73.68)	N/A

Serum 25(OH)D levels (median) (nmol/L)	45.0	22.0	0.001
IQR (Q1–Q3)	(33.0–65.0)	(15.0–31.0)	
25(OH)D deficiency (<20 nmol/L), *n* (%)	0 (0)	42 (44.2)	
25(OH)D insufficiency (20–40 nmol/L), *n* (%)	6 (31.6)	36 (37.9)	

On prednisolone, *n* (%)	8 (42.1)	Nil	N/A
<7.5 mg daily, *n* (%)	8 (42.1)		
≥7.5 mg daily, *n* (%)	0 (0)		

DMARDs, *n* (%)	15 (78.9)	Nil	N/A
On conventional DMARD, *n* (%)	0 (0)		
On anti-TNF therapy, *n* (%)	15 (78.9)		

Values are median and IQR, or numbers (*n*) and percentage (%) for counts. 25(OH)D: 25-hydroxyvitamin D; N/A: not applicable; IQR: interquartile range; Q1: 25th quartile; Q3: 75th quartile.

**Table 2:** Patients' clinical characteristics, treatment, and their vitamin D status.

Number	G	Age	OA	GU	OI	CI	PP	VI	GI	ENT	DA	25(OH)D	ESR	CRP
1	F	33	+	+	−	+	−	−	−	+	−	76	6	<5
2	M	51	+	+	+	+	+	−	+	+	−	71	7	7
3	F	35	+	+	−	+	−	−	−	+	A	42	25	5
4	F	23	+	+	−	+	−	−	−	+	−	45	11	<5
5	F	17	+	+	+	+	−	−	−	−	−	50	6	<5
6	M	48	+	+	+	+	−	+	−	−	−	65	12	<5
7	M	56	+	−	+	+	−	−	−	+	−	58	2	<5
8	F	37	+	+	−	+	−	−	−	−	−	67	12	<5
9	F	38	+	+	−	−	+	−	−	−	−	41	8	<5
10	M	34	+	+	−	+	−	−	−	−	−	76	17	<5
11	F	37	+	+	−	+	−	−	−	−	−	33	5	<5
12	F	52	+	+	−	+	−	+	−	−	A	28	28	<5
13	F	81	+	+	+	+	−	−	−	−	−	41	14	<5
14	F	66	+	+	−	+	−	−	+	−	−	35	33	<5
15	M	45	+	+	−	−	−	+	−	−	−	29	7	<5
16	F	37	+	+	−	+	−	−	−	−	A	21	28	25
17	F	23	+	+	+	+	−	−	−	−	−	53	5	<5
18	F	21	+	+	−	+	−	−	−	−	−	26	13	<5
19	F	24	+	+	−	+	−	−	−	−	A	49	14	<5

G: gender; OA: oral aphthosis; GU: genital ulcers; OI: ocular involvement; CI: cutaneous involvement; PP: pathergy phenomenon; VI: vascular involvement; GI: gastrointestinal involvement; ENT: otolaryngeal involvement; DA: disease activity; A: active disease; 25(OH)D: 25-hydroxyvitamin D (nmol/L); ESR: erythrocyte sedimentation rate (mm/hour); CRP: C-reactive protein (mg/L).

**Table 3:** Comparison of 25(OH)D levels between patients with active and inactive Behçet's disease.

Variables	Active BD (*n* = 4)	Inactive BD (*n* = 15)	*p* value
Serum 25(OH)D levels (median) (nmol/L)	35.0	50.0	0.11
(IQR1–IQR3)	(21.0–42.0)	(35.0–67.0)	
25(OH)D deficiency (<20 nmol/L), *n* (%)	0 (0)	0 (0)	
25(OH)D insufficiency (20–40 nmol/L), *n* (%)	2 (50.0)	4 (26.7)	

**Table 4:** Odds ratio of 25(OH)D deficiency in cases versus controls.

Variables	Unadjusted OR (95% CI)	*p* value	Adjusted OR (95% CI)	*p* value
*Sex*				
Female versus male	2.44 (1.00, 6.00)	0.051	3.01 (1.1, 8.26)	0.032
*Group*				
Cases (with BD) versus controls	0.10 (0.03, 0.30)	<0.001	0.09 (0.03, 0.28)	<0.001
